# Lenalidomide Exposure and *TP53*-Mutated Myelodysplastic Syndromes/Neoplasms

**DOI:** 10.3390/curroncol33090506

**Published:** 2026-08-26

**Authors:** Bahga Katamesh, Rory M. Shallis

**Affiliations:** Department of Malignant Hematology, Moffitt Cancer Center, Tampa, FL 33612, USA

**Keywords:** lenalidomide, MDS, myelodysplastic syndrome, Revlimid, *TP53*

## Abstract

The benefits of lenalidomide, which leverages TP53-dependent apoptosis and promotion of megakaryocytic differentiation, in lower-risk MDS (LR-MDS) with del(5q) are well-established and drive its frequent use in clinical practice. In parallel, advances in molecular testing have elucidated the clear impact of *TP53* mutations on response to therapies and prognosis, with diversity in the latter informed by variant allele frequency (VAF) and/or predicted allelic state. In this review, we discuss the converging pre-clinical and clinical evidence that implicates *TP53* mutations not only as a driver of lenalidomide resistance in del(5q) LR-MDS, but as a source of selective clonal advantage under lenalidomide exposure. Lenalidomide-induced CK1α degradation triggers TP53-mediated apoptosis in wild-type clones, inadvertently favoring the survival and expansion of *TP53*-mutated cells. Clinically, this dynamic has been demonstrated across multiple studies, with rising *TP53* variant allele frequency and clonal evolution observed following treatment. These findings underscore the importance of baseline molecular assessment in patients with MDS and, in select cases, serial monitoring during therapy. Investigational approaches for del(5q) LR-MDS with the *TP53* mutation include GATA2-directed strategies aimed at restoring lenalidomide sensitivity, as well as immune-based therapies targeting the tumor microenvironment characteristic of *TP53*-mutated disease.

## Simple Summary

Lenalidomide is a standard treatment for patients with lower-risk myelodysplastic syndromes/neoplasms (MDS) with isolated deletion of the long arm of chromosome 5 (del [5q]). Some cases also harbor mutations in *TP53*, a gene which has pleiotropic function and classically associates with poor response/outcome in other disease subtypes. There are conflicting data on the impact of lenalidomide in lower-risk del(5q) MDS with *TP53* mutation. This review synthesizes the available pre-clinical data for the influence of *TP53* mutation on lenalidomide function and the mechanisms by which these can evade its clinical effects. Baseline and/or serial molecular monitoring requires further study, as does means to mitigate if not abrogate the impact of *TP53* mutations in lower-risk del(5q) MDS.

## 1. Introduction

Myelodysplastic syndrome/neoplasm (MDS) with isolated deletion of the long arm of chromosome 5 (del [5q]) is a distinct subtype of MDS, comprising 10–20% of cases [1,2]. Del(5q) predicts loss of genes including *CSNK1A1*, *RPS14*, *MIR145* and *MIR146A* [3], all of which play important roles in myelodysplastic hematopoiesis. The 2022 World Health Organization (WHO) and International Consensus Classification (ICC) define del(5q) MDS as that with an isolated del(5q) or with one additional abnormality excluding a deletion in chromosome 7 or 7q, complex karyotype, a marrow/blood blast enumeration ≥ 5%, and bi-allelic *TP53* mutation [4]. Del(5q) MDS classically presents with a female predominance, a deep macrocytic anemia, normal or increased platelets, *SF3B1*/*DNMT3A*/*TET2* co-mutation, and ultimately a generally favorable prognosis [3].

Lenalidomide is approved as a first-line treatment for patients with transfusion-dependent del(5q) MDS. This was based on two pivotal studies, MDS-003 and MDS-004, that evaluated lenalidomide 10 mg daily amongst patients with International Prognostic Scoring System (IPSS) lower-risk MDS, including del(5q) MDS, and found an approximately 65% rate of red blood cell (RBC) transfusion independence (TI), 2.2-year duration of response, 72% rate of cytogenetic response, and lower rate of progression to AML, supporting a disease-modifying effect [5,6]. These data have also supported the National Comprehensive Cancer Network (NCCN) recommendation for lenalidomide as the first-line option for patients with lower-risk del(5q) MDS. Furthermore, the SintraREV trial recently demonstrated that earlier exposure with the lower dose of 5 mg daily amongst non-RBC transfusion-dependent patients with del(5q) MDS is associated with a striking rate/duration of RBC-TI, prompting consideration for such as an emerging standard [7]. Despite this success, approximately 40% of patients with isolated del(5q) MDS will have disease progression to acute myeloid leukemia (AML) within five years of starting treatment with lenalidomide [8]. In the post-authorization safety study by Poloni et al., the 24-month incidence of AML progression among 296 patients (including 277 who received at least one complete cycle of lenalidomide) was 12.7% [9]. The study included patients with transfusion-dependent IPSS low or intermediate-1 risk MDS with isolated del(5q). Treatment-related adverse events led to discontinuation in 35.5% of patients only, supporting the established benefit–risk profile of lenalidomide in this population.

**Figure 1:**
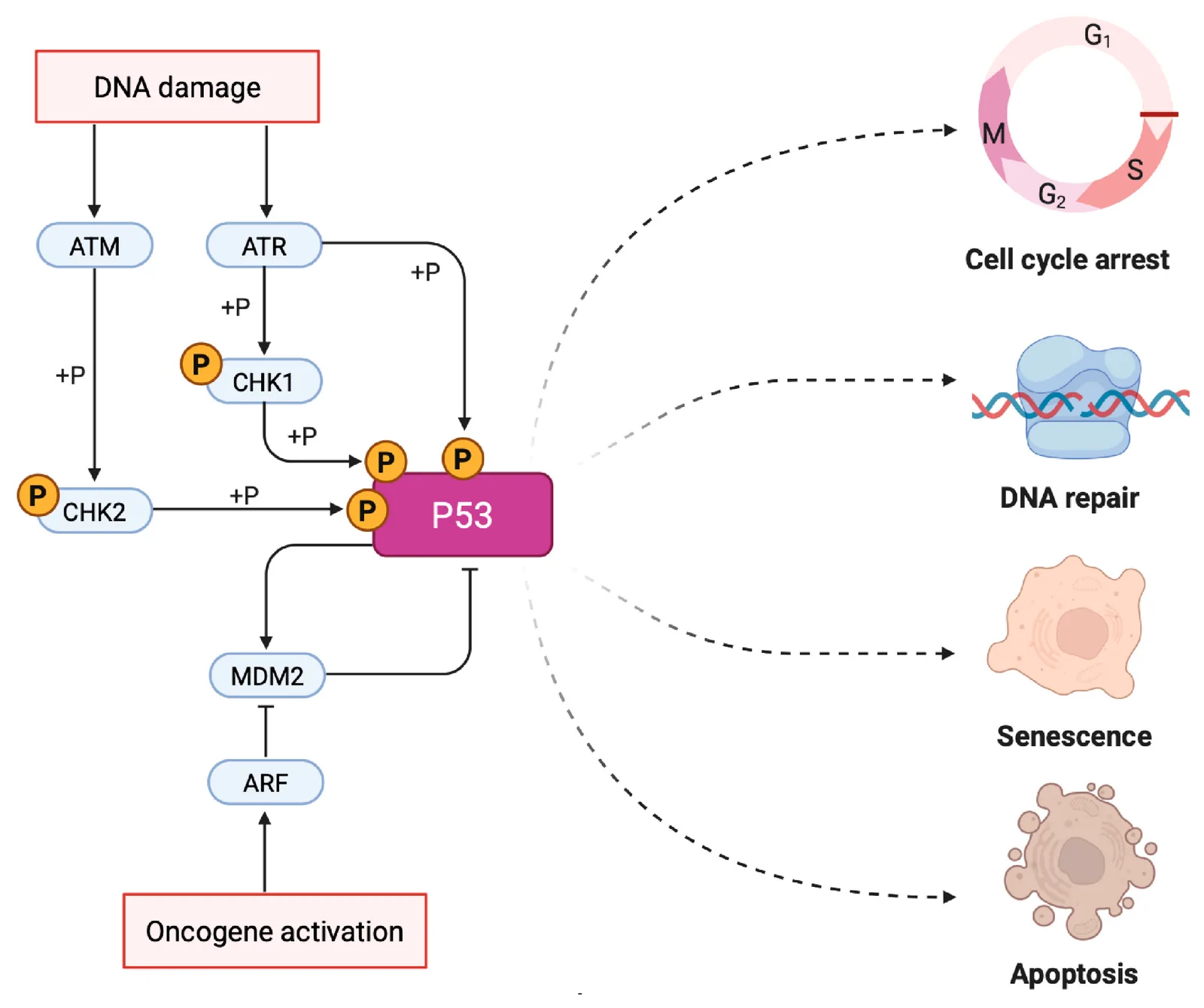
Activation and downstream effects of the TP53/p53 pathway. DNA damage activates the sensor kinases ATM and ATR, which phosphorylate the effector kinases CHK2 and CHK1, respectively. These kinases, together with direct ATR-mediated phosphorylation, activate p53 through post-translational phosphorylation. Oncogene activation provides a parallel input by inducing ARF, which inhibits MDM2, the principal negative regulator that otherwise targets p53 for degradation. Activated p53 orchestrates cell cycle arrest, DNA repair, senescence, and apoptosis, thereby preserving genomic integrity and preventing propagation of damaged cells.

This observation likely stems from the understanding that del(5q) MDS is still a biologically heterogeneous disease subtype and there are particular subgroups for whom lenalidomide exposure may predict differential response and outcome. *TP53* encodes the protein p53, a transcription factor known as the “guardian of the genome”. Once activated, it regulates the expression of hundreds of genes responsible for cell cycle regulation, apoptosis and DNA repair [10]. Thus, when impacted by loss of function, the mutation represents a common mechanism of both treatment resistance and progression in MDS (Figure 1). *TP53* mutations are detected in 18–24% of del(5q) MDS, and at a rate higher than that observed in non-del(5q) MDS [11]. Approximately 75% of these cases will harbor predicted *TP53* mono-allelic inactivation, with the remainder being “multi-hit” or associating with bi-allelic TP53 inactivation, with the latter being defined by concomitant loss of 17p (predicted by variant allele frequency [VAF] ≥ 50%, more than one distinct mutation or a *TP53* mutation with loss of heterozygosity), associating with complex karyotype, fewer co-mutations, and ultimately poor response to therapy and survival outcome; conversely, mono-allelic *TP53* inactivation predicts an outcome akin to *TP53* wild-type disease [12,13].

Pre-clinical and emerging clinical data implicate a lenalidomide-associated selective advantage to *TP53*-mutated clonal expansion, including amongst patients with del(5q) MDS, and invokes a relevant concern that this standard of care may not be such for all patients and, at a minimum, may beget variance in treatment and monitoring practices. In this review, we highlight the current pre-clinical and clinical data on the use of lenalidomide in *TP53*-mutated del(5q) MDS and discuss its clinical implications.

## 2. Lenalidomide Mechanism of Action and Interaction with TP53

**Figure 2:**
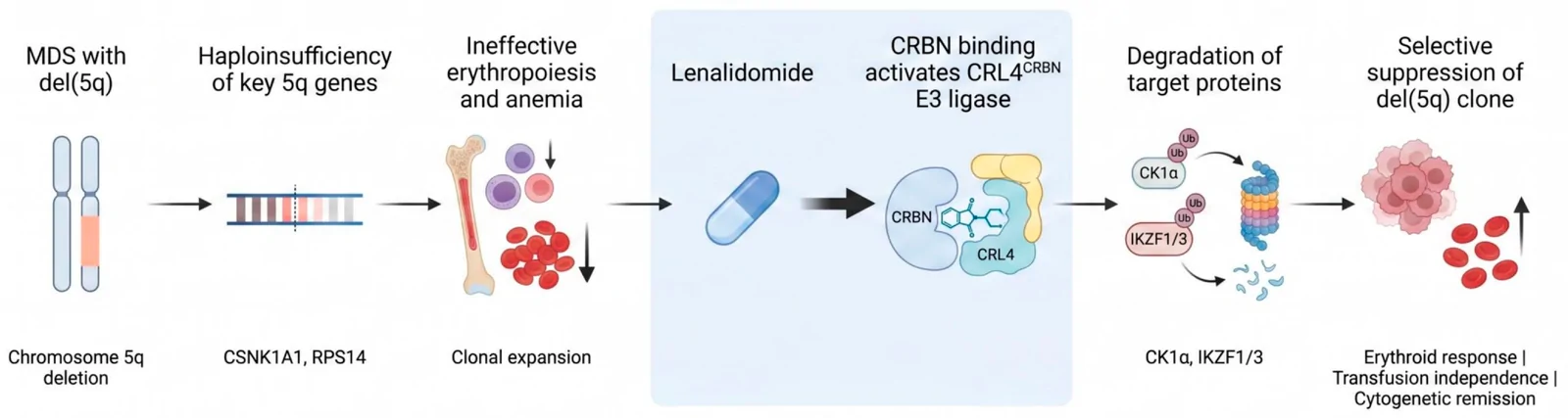
Mechanism of action of lenalidomide in del(5q). Haploinsufficiency of chromosome 5q genes, including *CSNK1A1* and *RPS14*, drives ineffective erythropoiesis and clonal expansion of the del(5q) clone. Lenalidomide binds cereblon (CRBN), a substrate receptor of the CRL4^CRBN^ E3 ubiquitin ligase complex, redirecting its activity toward neosubstrates CK1α and IKZF1/3. Subsequent degradation of these proteins selectively suppresses the del(5q) clone.

Lenalidomide is a derivative of the immunomodulator thalidomide that binds to cereblon [CRBN] [14], altering substrate specificity of CUL4-RBX1-DDB1-CRBN (CRL4^CRBN^) E3 ubiquitin ligase and promoting degradation of neo-substrates including casein kinase 1α (CK1α) [15], whose gene is located within the deleted segment of 5q. Consequently, del(5q) MDS is associated with a haploinsufficiency that promotes a selective vulnerability to lenalidomide-induced CK1α depletion [16]. The downstream effect after CK1α depletion amongst these clones is mediated through TP53-dependent apoptosis, with *TP53* mutations ultimately attenuating the cytotoxic mechanism that contributes to lenalidomide activity and efficacy [17] (Figure 2).

Furthermore, lenalidomide usually induces megakaryocytic differentiation of del(5q) cells through binding to CRBN, inducing degradation of IKZF1, which in turn leads to the formation of a RUNX1-GATA2 complex that drives differentiation [18]. The megakaryocytic differentiation in turn triggers calpain activation and CK1α degradation, leading to cell death [18]. *TP53* mutations promote a disruption of TP53-dependent upregulation of RUNX1, thereby conferring resistance [18]. Hence, loss-of-function *TP53* mutations or downstream underexpression of RUNX1 disrupts the pathway by which lenalidomide exerts its effect [18]. This was confirmed in a study by Martinez-Hoyer et al. in which overexpression of GATA2 was demonstrated to restore lenalidomide sensitivity via the central role of the RUNX1-GATA2 complex in driving megakaryocytic differentiation [18]. These data support the contention that overexpression of GATA2 restores sensitivity to lenalidomide even in the setting of *TP53* or *RUNX1* mutations. In addition, it has been well established that the *TP53*-mutated disease marrow is one characterized by distinct immune dysregulation, namely microenvironmental inflammation, immunosuppression via regulatory T-cell and myeloid-derived suppressor cell aberrancy, and impaired antigen presentation, all of which likely contribute to the therapeutic resistance observed with this molecular subtype [19,20].

**Figure 3:**
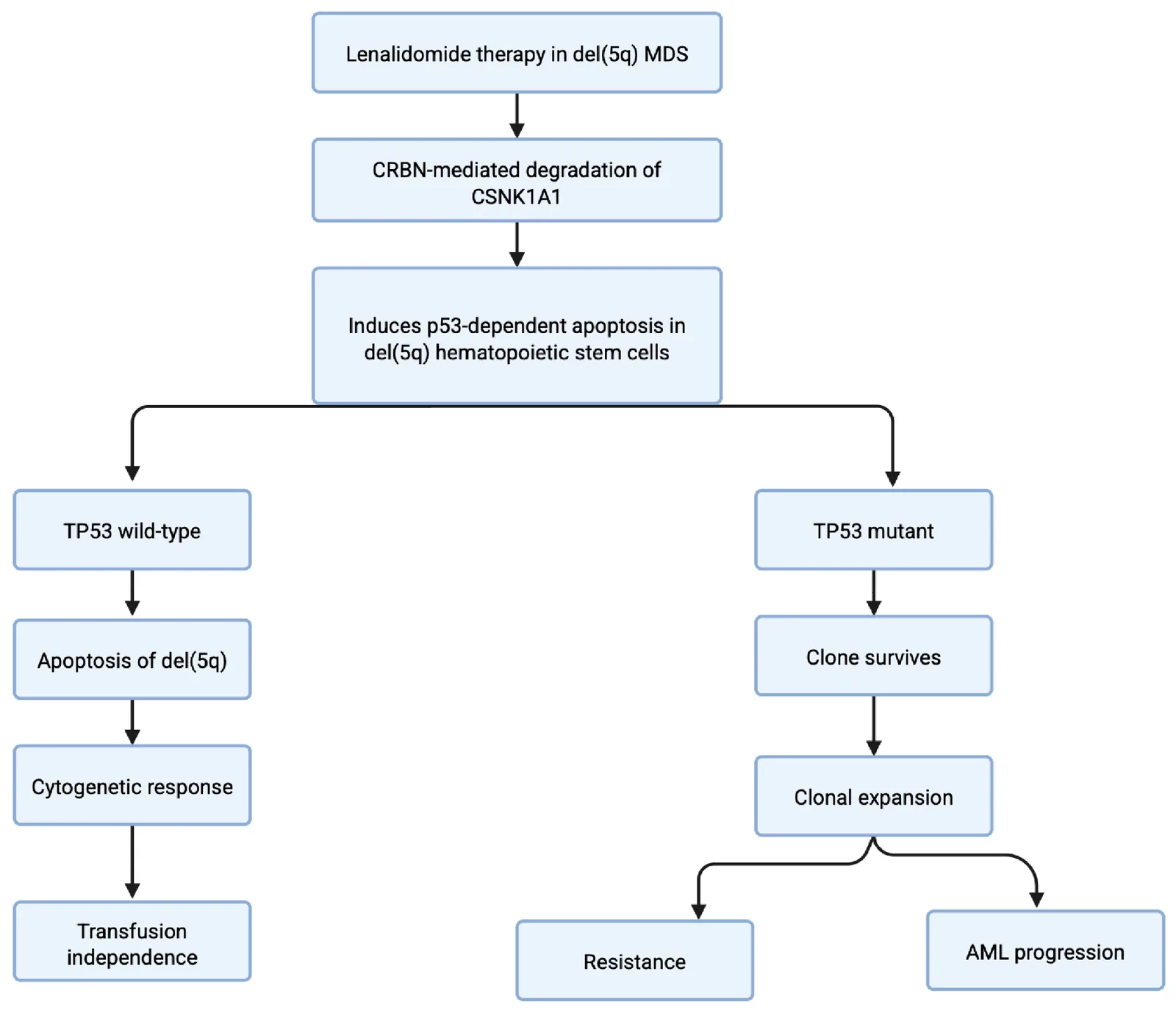
TP53 mutational status determines clonal outcome following lenalidomide therapy in del(5q) MDS. Lenalidomide promotes CRBN-mediated degradation of CSNK1A1, which induces p53-dependent apoptosis in del(5q) hematopoietic stem cells. In *TP53* wild-type disease, this leads to effective apoptosis of the del(5q) clone, cytogenetic response, and transfusion independence. In *TP53*-mutant disease, however, impaired p53 function allows the clone to evade apoptosis and survive, resulting in clonal expansion that drives either treatment resistance or progression to acute myeloid leukemia (AML).

Based on these mechanisms, it is hypothesized that lenalidomide allows a relative *TP53*-mutant clonal expansion. Sperling et al. in their analysis of 416 patients with therapy-related myeloid neoplasms found that *TP53* mutations were associated with prior lenalidomide exposure and that this association was specific to lenalidomide amongst the immunomodulatory drugs (positing a relation to CK1α degradation) [21]. This effect has also been demonstrated in vitro when hematopoietic stem cell progenitor cells are co-cultured either in the presence or absence of lenalidomide, with the former resulting in expansion of *TP53*-mutated cells compared to wild-type cells, as well as in mouse models through which lenalidomide expanded the TP53-mutated cell population [21]. Tehranchi et al. demonstrated that even during complete cytogenetic remission on lenalidomide, there remains a CD34+ CD38−/low CD90+ population of quiescent stem cells that harbor del(5q) [22]. It is proposed that this population is what drives relapse in these patients. In the same sense, *TP53* mutations harbored in these cells would eventually drive transformation to AML. In other mouse models, it was found that the cooperative effect of TP53 loss and haploinsufficiency of *EGR1* and *APC2* genes—usually found in the 5q deletion region—creates a permissive environment for transformation [23] (Figure 3).

## 3. TP53 Mutations and the Clinical Impact of Lenalidomide Exposure

*TP53* status does indeed inform the possibility of differential del(5q) pathobiology and clinical outcome. Some of the earlier studies that unraveled the role of TP53 in del(5q) MDS include studies by Jadersten et al. and Mallo et al. [24,25]. Jadersten and colleagues studied 55 patients with low/intermediate-risk MDS by IPSS. *TP53* mutation was found in 10 patients prior to treatment and was associated with progression to AML (5 of 10 compared to 7 of 45, *p* = 0.045). These patients also had a lower rate of complete cytogenetic response to lenalidomide (*p* = 0.024) [24]. Mallo demonstrated that in 52 del(5q) MDS patients treated with lenalidomide, none of those with *TP53* mutations showed a complete cytogenetic response, while those with unmutated *TP53* tended to show a hematologic response (*p* = 0.061). In addition, *TP53* mutations emerged in 38% of patients a median of 11 months post-exposure to lenalidomide [25].

When dichotomized by the presence of complex or non-complex karyotype, the frequency of *TP53* mutations amongst patients with del(5q) MDS is shown to be higher with complex karyotype (81% versus 27%) [26]. Unlike isolated del(5q) MDS where the ancestral event is predicted to be del(5q), *TP53* mutations are believed to be amongst the founding lesions in del(5q) MDS with complex karyotype. These data, in addition to the data in the forthcoming clinical discussion, support the 2022 WHO and ICC positions that MDS with biallelic *TP53* inactivation, even when occurring with what would otherwise be classified as MDS del(5q), should be considered a distinct entity [4]. Some of the above studies are summarized in Table 1.

The largest evaluation of *TP53*-mutated del(5q) MDS to date was performed by Montoro and colleagues [13]. In their study of 682 cases of del(5q) MDS, approximately 19% were identified as also harboring a pathogenic variant in *TP53*, with 76% of such predicted as being mono-allelic *TP53* inactivation. The median survival of patients with *TP53*-mutated del(5q) MDS was strikingly favorable with a 73-month median overall survival (OS) demonstrated for patients with mono-allelic *TP53* inactivation, mirroring the 77 months for patients with *TP53* wild-type disease. However, *TP53* bi-allelic inactivation was found to associate with an increased risk of secondary AML and shorter OS (57 months) when compared with these two subgroups. An updated analysis including an additional 12 cases of *TP53*-mutated del(5q) MDS affirmed these findings [27]. Amongst the 294 patients who were treated with lenalidomide, *TP53* mutations did associate with a lower rate of response when compared with wild-type disease but did not achieve statistical significance (61% vs. 74%, *p* = 0.07), and allelic state predicted no difference (57% for mono-allelic, 75% for bi-allelic; *p* = 0.30). Thus, the authors hypothesized that *TP53* bi-allelic inactivation may not exert the same clinical impact that is observed classically amongst patients in the larger MDS population, and a revision to the current classification system isolating all MDS with bi-allelic *TP53* inactivation irrespective of del(5q) status may be in order. Similarly, Tefferi et al. analyzed 210 cases of del(5q) MDS [28], including a number of cases that were also included in the analyses by Montoro and colleagues [13], and identified a *TP53* VAF > 22% to be independently associated with both inferior leukemia-free survival (LFS) and OS. In fact, del(5q) MDS with VAF < 22% and not qualified as being “therapy related” was associated with a median OS of nearly 12 years, compared with the 4-year median OS of their counterparts. This suggests that continuous VAF measurement may identify clinical heterogeneity that is not fully disclosed by the binary mono/bi-allelic classification of *TP53* inactivation. Most recently, these groups pooled data with other centers to derive a total of 43 patients with MDS with isolated del(5q) and compared the group to 68 patients with MDS-LB with multi-hit *TP53* but without isolated del(5q) [27]. The group ultimately affirmed that patients with del(5q) MDS with multi-hit *TP53* had a longer time of progression to AML (31.9 versus 7.2 months) and longer median OS (70.2 months vs. 13.9 months) when compared with patients with MDS-LB with multi-hit *TP53* but without concurrent del(5q). The superior outcome persisted even when MDS del(5q) multi-hit *TP53* was compared to MDS-LB without complex karyotype. These data suggest that multi-hit inactivation of TP53 in the context of del(5q) may be less clinically relevant than in other MDS subtypes. Of note, these studies did not evaluate the effect of lenalidomide exposure on serial *TP53* VAF, to isolate patients for whom the outcome may not be as uniform.

The SintraREV trial, which studied lenalidomide 5 mg daily for non-RBC-transfusion-dependent patients with del(5q) MDS, included five patients who had *TP53*-mutated disease, and did indeed evaluate serial *TP53* VAF on treatment [7]. The analysis revealed that patients with *TP53*-mutated del(5q) MDS were exclusively those with *TP53* mono-allelic disease and revealed VAF reductions of ≥10% across all mutations, suggesting disease modification that would be expected with lenalidomide exposure in del(5q) MDS overall. In addition to these limited analyses, the study revealed shorter LFS amongst patients with *TP53*-mutated disease, as well as lower erythroid response and cytogenetic response rates when compared with patients with *TP53* wild-type disease. A molecular sub-study of the trial (including 51 patients) also showed that early exposure to lenalidomide reduced the overall mutational burden and did not promote expansion of *TP53* clones [29]. In this study by Castello et al., the most frequently found mutations were *SF3B1* (25.5%) and *TP53* (21.3%). Both decreased or remained stable under lenalidomide, versus increasing or remaining stable in 100% of placebo patients at 2 years.

Taken together, the data suggests that the influence of *TP53* mutations on lenalidomide response is best interpreted as a gradient rather than a binary of mutated versus wild-type. The data suggests that the presence of the alteration blunts cytogenetic response. Allelic state also has an impact, with the majority of disease being mono-allelic and having comparable outcomes to wild-type *TP53* disease, but current studies suggest that multi-hit alterations in the context of del(5q) behave less aggressively than those outside of del(5q). VAF is the most granular discriminator of outcome, and serial VAF measurement may be clinically relevant in predicting disease trajectory.

## 4. Clinical Recommendations and Future Directions

Current NCCN guidelines recommend treating isolated del(5q) MDS with lenalidomide, representing the preferred standard frontline treatment for symptomatic/actionable anemia. However, appropriate consideration is recommended for the patients with del(5q) with *TP53* mutation, namely that for “additional consideration for and/or closer monitoring of particular patients is warranted” in addition to the larger MDS population-centric statement that patients with *TP53*-mutated disease, particularly biallelic, have a poor prognosis even with allogeneic hematopoietic stem cell transplantation (alloHCT), with patients recommended to be managed as high-risk regardless of any other clinical features of disease and enrolled in a clinical trial whenever possible. Serial monitoring of *TP53* VAF may be a consideration for patients with del(5q) MDS patients exposed to lenalidomide and upon relapse/progression.

Novel therapies for MDS are required to further the incremental improvement in outcomes seen with contemporary management, particularly for patients with *TP53*-mutated disease, and perhaps could be studied in combination with standard-of-care approaches, even del(5q) MDS, despite the relatively favorable outcomes associated with lenalidomide. Newer approaches to the management of TP53-mutated MDS del(5q) include the use of combination therapies, including the combination of non-immune and novel immune strategies. However, Pereira et al. argue that while some of these emerging combinations have shown improved overall response rates, it has not yet translated to an improved survival [30].

Several novel therapies have been studied specifically for *TP53*-mutated disease, although many have failed in the clinical setting, including in randomized phase 3 trials [31]. Focus could also be directed towards the targeting of lenalidomide resistance pathways. Inhibition of IGF-1R, which is upregulated in del(5q) MDS, reduces the proliferation of resistant del(5q) cells both in vivo and in vitro [32]. Other mechanisms of action are being explored with several other novel agents entering early phase clinical study. The role of alloHCT, the only curative option for MDS, upon a diagnosis of del(5q) MDS with *TP53* mutation, including that with predicted bi-allelic inactivation, remains controversial and should be individualized based on a particular patient’s clinical situation.

## 5. Conclusions

Lenalidomide remains the standard of care for patients with transfusion-dependent MDS del(5q), even in the presence of a *TP53* mutation, despite the aforementioned data on the resistance of *TP53*-mutated clones to lenalidomide and a possible selective advantage conferred by exposure. The bulk of the clinical literature supports no marked detriment to survival, but the nuance of lenalidomide dosing, co-mutation, and VAF kinetics requires more study.

## Figures and Tables

**Table 1:** Clinical studies evaluating lenalidomide outcomes in del(5q) MDS stratified by TP53 mutation status.

Study	N (Total; TP53-Mutated)	TP53 Stratification	TP53 Wild-Type Outcome	TP53-Mutant Outcome	Ref
Jädersten et al.	55 (n = 10 TP53-mutated)	Mutation present vs. absent (not allelic state)	AML progression: 7/45 (16%)	AML progression: 5/10 (50%), *p* = 0.045 Lower complete cytogenetic response, *p* = 0.024	[24]
Mallo et al.	52 del(5q) MDS treated with lenalidomide	Mutation present vs. absent	Trend toward hematologic response, *p* = 0.061	0% complete cytogenetic response TP53 mutations emerged in 38% of patients (median, 11 months post-exposure)	[25]
Montoro et al. (largest cohort to date)	682 del(5q) MDS (19% TP53-mutated; 76% mono-allelic)	Mono-allelic vs. bi-allelic vs. wild-type	Median OS: 77 months	Mono-allelic: median, OS 73 months (mirrors wild-type) Bi-allelic: median OS, 57 months; higher risk of secondary AML	[13]
Updated Montoro cohort (lenalidomide-treated subset)	294 lenalidomide-treated	Mutated vs. wild-type; mono- vs. bi-allelic	Response rate: 74%	Response rate: 61% (*p* = 0.07, not significant) Mono-allelic 57% vs. bi-allelic 75% (*p* = 0.30, not significant)	[27]
Tefferi et al.	210 del(5q) MDS	TP53 VAF > 22% vs. < 22%	VAF < 22%, non-therapy-related: median OS ~12 years	VAF > 22%: independently associated with shorter leukemia-free survival and OS (~4-year median OS)	[28]
Pooled analysis (multi-hit TP53, del(5q) vs. non-del(5q))	43 del(5q) MDS multi-hit TP53 vs. 68 MDS-LB multi-hit TP53 without del(5q)	Multi-hit TP53 with vs. without concurrent del(5q)	Not applicable (comparison restricted to TP53-mutant cases)	With del(5q): time to AML, 31.9 months; median OS, 70.2 months Without del(5q): time to AML, 7.2 months; median OS, 13.9 months	[27]
SintraREV trial (TP53-mutated subgroup)	n = 5 TP53-mutated (all mono-allelic); total trial N not specified in manuscript text	Mono-allelic mutated vs. wild-type	Higher erythroid and cytogenetic response rates; longer leukemia-free survival	>10% VAF reduction observed across all mutations on treatment Lower erythroid and cytogenetic response rates; shorter leukemia-free survival	[7]

Abbreviations: AML, acute myeloid leukemia; MDS, myelodysplastic syndrome; MDS-LB, MDS lower blast; OS, overall survival; VAF, variant allele frequency. The pivotal MDS-003/MDS-004 trials establishing lenalidomide as first-line therapy for del(5q) MDS (refs. [5,6]) are not included, as these predate routine TP53 mutation testing and did not stratify outcomes by TP53 status.

## Data Availability

No new data were created or analyzed in this study. Data sharing is not applicable to this article.
